# Changing climate mediates sapsucker (Aves: *Sphyrapicus*) hybrid zone movement

**DOI:** 10.1002/ece3.2507

**Published:** 2016-10-12

**Authors:** Shawn M. Billerman, Melanie A. Murphy, Matthew D. Carling

**Affiliations:** ^1^ Department of Zoology and PhysiologyUniversity of WyomingLaramieWYUSA; ^2^ Program in EcologyUniversity of WyomingLaramieWYUSA; ^3^ Department of Ecosystem Science and ManagementUniversity of WyomingLaramieWYUSA

**Keywords:** climate change, climatic niche, hybrid zone, movement, sapsucker, species distribution modeling

## Abstract

Hybrid zones, where two divergent taxa meet and interbreed, offer unique opportunities to investigate how climate contributes to reproductive isolation between closely related taxa and how these taxa may respond to climatic changes. Red‐naped (*Sphyrapicus nuchalis*) and Red‐breasted (*Sphyrapicus ruber*) sapsuckers (Aves: Picidae) hybridize along a narrow contact zone that stretches from northern California to British Columbia. The hybrid zone between these species has been studied extensively for more than 100 years and represents an excellent system for investigations of the evolution of reproductive isolation. Shifts in the proportions of phenotypes at hybrid localities since 1910 that were inferred using specimens from museum collections were confirmed using species distribution models. We predicted the historical, current, and future distributions of parental and hybrid sapsuckers using Random Forests models to quantify how climate change is affecting hybrid zone movement in the Pacific Northwest. We found observed distribution shifts of parental sapsuckers were likely the result of climate change over the past 100 years, with these shifts predicted to continue for both sapsuckers over the next 80 years. We found Red‐breasted Sapsuckers are predicted to continue to expand, while Red‐naped Sapsuckers are predicted to contract substantially under future climate scenarios. As a result of the predicted changes, the amount of overlap in the distribution of these sapsuckers may decrease. Using hybrid phenotypes, we found the climate niche occupied by the hybrid zone is predicted to disappear under future conditions. The disappearance of this climate niche where the two parental species come into contact and hybridize may lead to a substantial reduction in genetic introgression. Understanding the impacts of global climate change on hybrid zones may help us to better understand how speciation has been shaped by climate in the past, as well as how evolution may respond to climate change in the future.

## 1 Introduction

The processes that generate biodiversity are inextricably linked with climate. As such, climate can be a strong predictor of species distributions (Pearson & Dawson, 2003), impacting species either directly through physiological tolerance (e.g., Pörtner, 2002) or indirectly through community interactions such as habitat suitability (e.g., Hirzel & Lay, 2008). Climate also strongly impacts where divergent taxa may meet in secondary contact and form hybrid zones (Swenson & Howard, 2005; Swenson, 2006). Hybrid zones thus represent good systems to study the potential effect of climate on patterns of species divergence, as climate can influence the strength of different isolating barriers by impacting the degree to which hybridizing taxa overlap and interact (Carling & Thomassen, 2012; Carling & Zuckerberg, 2011; Engler, Rödder, Elle, Hochkirch, & Secondi, 2013; Grant & Grant, 2002; Nosil, Harmon, & Seehausen, 2009; Schemske, 2010; Taylor, Larson, & Harrison, 2015; Taylor et al., 2014).

Stable hybrid zones have long been used to investigate the factors contributing to reproductive isolation (e.g., Barton & Hewitt, 1985, 1989; Brelsford & Irwin, 2009; Sætre & Sæther, 2010; Voros, Alcobendas, Martínez‐Solano, & García‐París, 2006). More recently, it has become clear that unstable (i.e., moving) hybrid zones can be fruitful systems for understanding the evolution of reproductive isolation under unstable, changing scenarios (e.g., Buggs, 2007; Engebretsen, Barrow, Rittmeyer, Brown, & Lemmon, 2016; Hairston, Wiley, Smith, & Kneidel, 1992; Pearson & Rohwer, 2000; Taylor et al., 2015; McDonald et al., 2001). Shifts in hybrid zones may be due to differential population densities of parental species (Barton & Hewitt, 1985, 1989; Buggs, 2007), adaptive differences (Barton & Hewitt, 1985), selection (e.g., Dasmahapatra et al., 2002), and environmental change (Buggs, 2007; Carling & Zuckerberg, 2011; Hairston et al., 1992; Taylor et al., 2014, 2015; Walls, 2009). Furthermore, it is apparent that distribution shifts of hybridizing taxa due to environmental change can be the result of either physical change to the habitat (e.g., logging—Haig, Mullins, Forsman, Trail, & Wennerberg, 2004) or climate‐mediated change (Carling & Zuckerberg, 2011; Chunco, 2014; Engler et al., 2013; Garroway, Bowman, Holloway, Malcolm, & Wilson, 2011; Taylor et al., 2014, 2015).

Climate‐mediated change, whereby species distributions shift in response to changing climate conditions, can influence hybrid zones in two main ways: (1) the contact zone may shift with a changing climate as new climatic conditions favor one species over another (Carling & Zuckerberg, 2011; Taylor et al., 2014, 2015) or (2) new hybrid zones may form as a result of climate change, as previously isolated taxa come into secondary contact following climate‐driven range expansion (Chunco, 2014; Garroway et al., 2010, 2011). Moving hybrid zones represent excellent systems to study the impacts of climate change on speciation and the evolution of reproductive isolation, because they afford us an opportunity to determine what in a system has changed and how that change influences reproductive isolation (Buggs, 2007; Taylor et al., 2015). Interestingly, climate‐driven hybrid zone change has been documented over a wide time scale, ranging from hundreds to thousands of years (e.g., Ruedi, Smith, & Patton, 1997), to just decades (e.g., Engebretsen et al., 2016; Walls, 2009).

**Figure 1:**
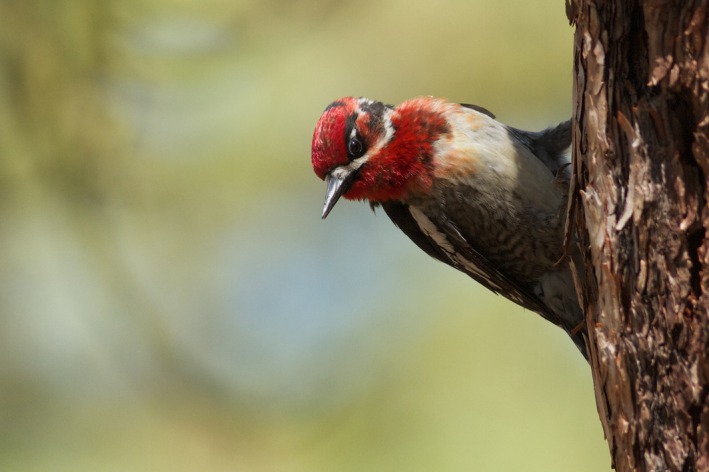
Red‐breasted (*Sphyrapicus ruber*) × Red‐naped sapsucker (*Sphyrapicus nuchalis*) hybrid. Photographed in Fremont National Forest, Lake County, Oregon, May 2013.Photograph used with permission and copyright Thomas B. Johnson

In this study, we investigate how climate is contributing to movement of an avian hybrid zone. Two species of North American sapsucker (Aves: *Sphyrapicus*) form an exemplary system to investigate climate‐driven hybrid zone shifts. Red‐naped (*Sphyrapicus nuchalis*) and Red‐breasted sapsuckers (*Sphyrapicus ruber*) hybridize over a narrow contact zone that stretches from British Columbia to northern California. Within this narrow contact zone where the ranges of these two species overlap, individuals with hybrid phenotypes are frequently encountered (Figure 1; Cicero & Johnson, 1995; Howell, 1952; Johnson & Johnson, 1985; Trombino, 1998). Across the Pacific Northwest region of North America where these two species breed, Red‐naped Sapsuckers are migratory to portions of the southwestern United States, including California, Arizona, New Mexico, and parts of Mexico (Walters, Miller, & Lowther, 2014a). Red‐breasted Sapsuckers in the region are represented by both migratory and sedentary populations: migratory birds largely breed east of the crest of the Cascade Mountains and spend the winter in California, while sedentary populations are found closer to the coast (Walters, Miller, & Lowther, 2014b). Where Red‐breasted Sapsuckers are migratory, they have been found to arrive on the breeding grounds about 2 weeks earlier than Red‐naped Sapsuckers (Trombino, 1998).

We take advantage of a dataset within this hybrid zone that spans over 100 years. Museum collections allow us to determine the exact position of the hybrid zone across multiple time points and examine how shifts have occurred over decades, as well as the role climate plays in shaping the distribution of hybridizing taxa through time. Based on museum collections, we can detect drastic shifts in the proportions of the two parental species and their hybrids at specific sites. In the Warner Mountains of northeast California and south‐central Oregon, sapsucker populations have steadily shifted: in the early 1900s, only Red‐naped Sapsuckers were detected (Howell, 1952; Johnson & Johnson, 1985), although by the mid‐1900s through the 1990s, both Red‐naped and Red‐breasted sapsuckers, and their hybrids, were found in high proportions (Johnson & Johnson, 1985; Trombino, 1998). Recent museum collections from 2012 show birds with predominantly Red‐breasted Sapsucker phenotypes with some hybrids, but no Red‐naped Sapsuckers (Billerman and Carling, in prep). Small‐scale shifts have also been found along the east flank of the Cascades in central Oregon, where Red‐breasted Sapsuckers were only found in low numbers at higher elevations in the early 1900s (Howell, 1952). Howell (1952) later found Red‐breasted Sapsuckers much farther downslope and in larger numbers where only Red‐naped Sapsuckers had been previously found. More recent evidence of movement in the sapsucker hybrid zone comes from the Ochoco Mountains in central Oregon, where, until recently (circa 2000), only Red‐naped Sapsuckers had been present (S. A. Shunk, personal communication). Recent work (2011–2013) suggests hybrid phenotypes are now regularly encountered (Billerman and Carling, in prep) in the Ochoco Mountains.

Despite strong evidence that the sapsucker hybrid zone has moved, the underlying mechanisms are unknown. Several hypotheses have been proposed to explain movement in the sapsucker hybrid zone, including (1) differential aggression between parental species, with Red‐breasted Sapsuckers being aggressively dominant over Red‐naped Sapsuckers (Billerman and Carling, in review) and (2) patterns of differential mate choice, where females of both species prefer males with more red, leading to the observed eastward expansion of Red‐breasted Sapsuckers (Johnson & Johnson, 1985). Here, we test an alternative hypothesis that climate is contributing to hybrid zone movement. Assessing the role of climate in the sapsucker system will help us to determine which factors may contribute most to the distribution of these two species and their hybrids. Therefore, our first objective is to define the climatic niche of parental and hybrid sapsuckers. Using these niches, we then seek to address the following questions: (1) How well do climate data predict the current distribution of sapsuckers and the region where they hybridize? (2) Is there evidence that past climate change has impacted the observed shifts in the distributions of parental species, thus impacting where they come into contact and might hybridize? and (3) Will future climate change lead to predicted movement of the Red‐breasted and Red‐naped sapsucker hybrid zone? We predict that climate has led to the expansion of Red‐breasted Sapsuckers and subsequent range retraction of Red‐naped Sapsuckers and that this is an ongoing process that will continue to drive similar distribution changes in this system.

## 2 Methods

### 2.1 Species occurrence

**Figure 2:**
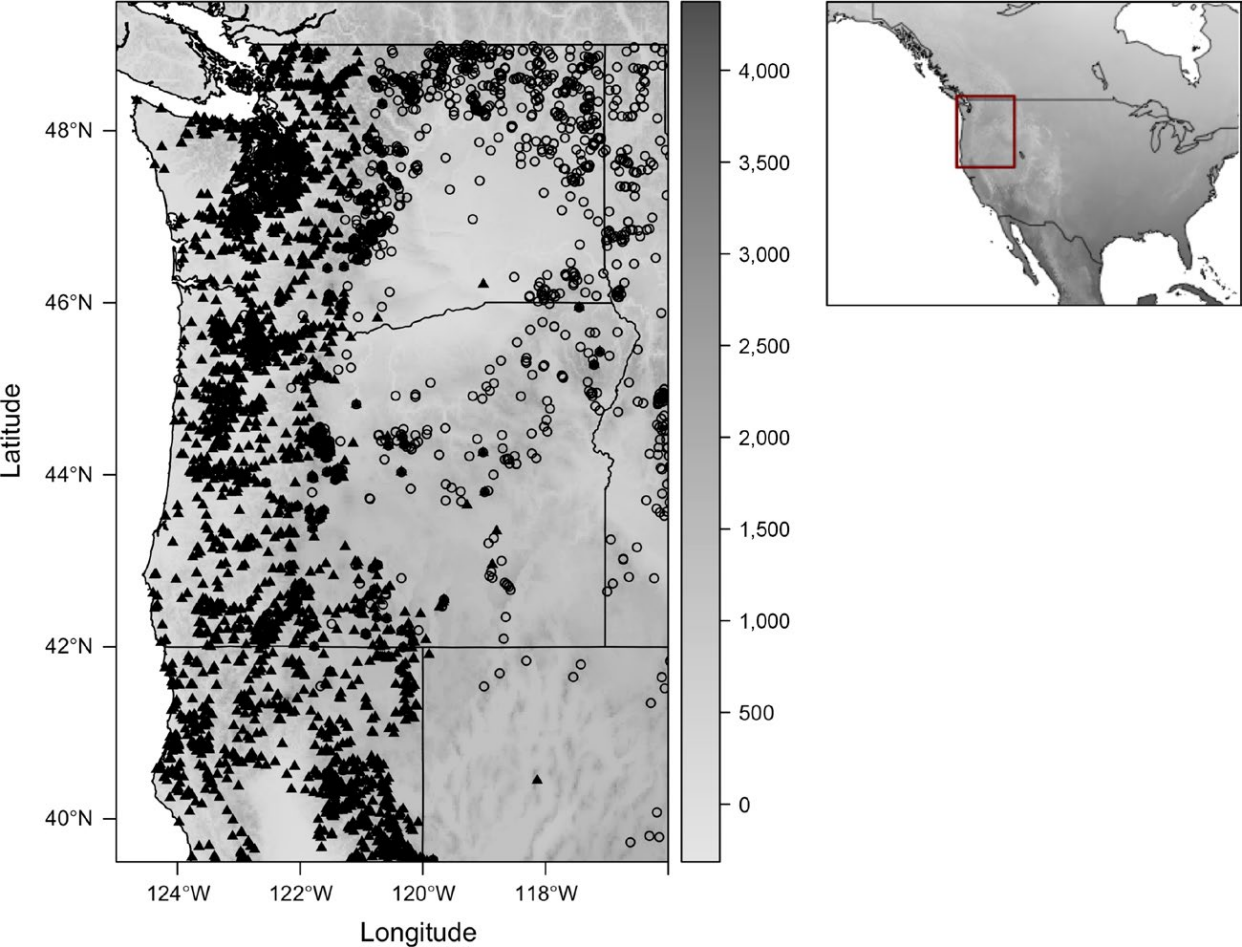
Map showing the localities for Red‐naped (*Sphyrapicus nuchalis*) and Red‐breasted Sapsuckers (*Sphyrapicus ruber*) used in current random forest models. Closed triangles represent Red‐breasted Sapsuckers, and open circles represent Red‐naped Sapsuckers. Inset shows the study area (red box) relative to the rest of North America. The scale (vertical bar) on the right side of the figure corresponds to elevation (meters)

Our study area in the Pacific Northwest included Washington, Oregon, northern California, and the western portion of Idaho and Nevada (USA; Figure 2). The extent of our study region was defined as follows: north boundary: 49.5°N, south boundary: 39.5°N, west boundary: 125°W, and east boundary: 114°W. We chose this as our extent to focus primarily on the southern portion hybrid zone between Red‐naped and Red‐breasted sapsuckers, where there has been a long history of research on sapsucker hybridization (Howell, 1952; Johnson & Johnson, 1985; Trombino, 1998). Species locality data for this region were obtained from three sources: the USGS North American Breeding Bird Survey Database (*n *=* *236 unique localities; Pardieck, Ziolkowski, & Hudson, 2014; Table S1), Project eBird (*n *=* *4,506 unique localities; Sullivan et al., 2009), and museum specimens (*n *=* *81 unique localities, Tables S2 and S3), for a total sample size of 4,824 presence localities (Tables S1–S3). We defined presence localities as any location for which a sapsucker was observed or collected (for museum specimens) at any point in time during the breeding season, which we here define as June and July (some specimens included were collected in May; these birds all showed signs of breeding, thus were likely not migrants). We accessed locality data for museum specimens housed at the University of Wyoming Museum of Vertebrates and the University of California at Berkeley Museum of Vertebrate Zoology using the online Arctos database (arctos.database.museum). Additionally, we accessed locality data for museum specimens housed at the University of Washington Burke Museum of Natural History and Culture directly from their online database (www.burkemuseum.org). Project eBird is a citizen science database that uses a network of over 400 regional reviewers to check the quality of the data submitted by observers (Fink et al., 2010; Sullivan et al., 2009). The data we used from eBird include observations made through August 2013. The earliest observation we used from the eBird database was from 1969. We used localities from eBird for which complete checklists existed to determine the presence or absence as in Fink et al. (2010): a species was considered to be absent when one species was found at this locality but the other was not. In this way, individual points could be a presence locality for one species while being an absence point for the other. To sample climate space that was unsuitable for either species of sapsucker, we obtained additional absence localities from a random subset of eBird checklists where neither species of sapsucker was observed (*n *=* *680). We used these localities to improve the predictive power of our species distribution models. For the current distribution models, we included species locality data from 1969 to 2013. For the historical distribution models, we pooled locality data collected between 1910 and 1940.

In addition to localities for each parental species, we also collected locality information from individuals identified as hybrids from eBird (*n *=* *59) and from museum specimens (*n *=* *54). During the breeding season, hybrid phenotypes are only encountered within the hybrid zone where the two parental species come into contact. We assume that localities with hybrid individuals represent part of the hybrid zone (Howell, 1952; Johnson & Johnson, 1985; Trombino, 1998). While hybrid phenotypes can be difficult to identify, in most places where they occur, hybrid entries to eBird are checked by regional reviewers, which ensures a relatively high quality of hybrid locality data used in our study. In order to be accepted by regional reviewers, these records would require either high‐quality photographs to establish the sighting or careful descriptions of individuals noted as hybrids, explaining the criteria used to separate hybrid individuals from parental species. These descriptions would have to match established field marks for separating these species based on previous research of the sapsucker hybrid zone (Howell, 1952; Johnson & Johnson, 1985; Trombino, 1998).

We calculated Ripley's *K* and nearest neighbor estimates (“spatstat”; Baddeley, Turner, & Rubak, 2016) in R (R Core Development Team, 2015) to test for significant clustering of our locality data (Fortin & Dale, 2005). We found that data from the eBird database were significantly clustered for both Red‐naped and Red‐breasted sapsucker observations (Figure S1). To correct for bias introduced by clustering of our data, where clustering may be the result of observers visiting popular birding locations, we randomly selected a single observation from an approximate 1‐square‐kilometer grid (0.015° longitude by 0.01° latitude). We found that the filtered dataset and the full, unfiltered dataset resulted in very similar models, with equivalent levels of support (Figure S2). We only show results from our full, unfiltered datasets.

### 2.2 Climate data

We acquired climate data from two sources: (1) the PRISM Climate Group (2013), which we used to extract both historical climate data and current climate data, and (2) the Moscow Forestry Sciences Laboratory (MFSL: Rehfeldt, Crookston, Warwell, & Evans, 2006), which we used to obtain current climate data and future climate scenarios. To develop historical climate surfaces, we created 30‐year (1910–1940) climate normals based on monthly interpolated conditions of precipitation, maximum temperature, minimum temperature, and mean annual temperature using data from the PRISM Climate Group (2013). From these four variables, we created five additional climate variables: December precipitation, July precipitation, precipitation seasonality, January minimum temperature, and July maximum temperature. We define precipitation seasonality as the degree of variability in monthly precipitation throughout the year and calculated this variable by calculating the coefficient of variation of precipitation across each year using the “raster” package (Hijmans, van Etten, & Cheng, 2015) as implemented in R (R Core Development Team, 2015). To directly compare between historical and current climate projections, we developed current climate surfaces using these same nine variables: we created 30‐year (1970–2000) climate normals based on monthly interpolated conditions of precipitation, maximum temperature, minimum temperature, and mean temperature using data from the PRISM Climate Group (2013). We used those four variables to calculate December precipitation, July precipitation, precipitation seasonality, January minimum temperature, and July maximum temperature (Table 1). We also tested 16 climate variables from the MFSL (Rehfeldt et al., 2006) for multicollinearity using the “rfUtilities” package (Evans & Murphy, 2014) as implemented in R (R Core Development Team, 2015). Using a multicollinearity threshold of 10^−7^, we removed six climate variables from our dataset. We included 10 nonredundant variables from the MFSL for current and future models (Table 2; Evans & Murphy, 2014; Evans, Murphy, Holden, & Cushman, 2011; Murphy, Evans, & Storfer, 2010). We included these particular variables (Tables 1 and 2) because they have been shown to be important in structuring the distribution of bird species (Peterson et al., 2007). Though we only modeled breeding distributions, we included variables related to winter conditions as winter climate influences forest dynamics of western North America where sapsuckers breed (Mantgem et al., 2008; Rehfeldt, Ferguson, & Crookston, 2009). Winter climate conditions can also influence migration time (Gienapp, Leimu, & Merilä, 2007; Hüppop & Hüppop, 2003; Stervander, Lindström, Jonzén, & Andersson, 2005) and the timing of breeding by affecting the period of peak insect abundance (Saino et al., 2011; Thomas, Blondel, Perret, Lambrechts, & Speakman, 2001), as well as affecting ecosystem productivity by altering year‐round water availability (Illán et al., 2014). We also included winter climate in our models because some populations of Red‐breasted Sapsuckers included in our study area are sedentary, and thus directly experience winter conditions (Walters et al., 2014b).

**Table 1:** Climate variables from the PRISM Climate Group (2013) used in Random Forests models for both current and historical classifications. Number represents variable importance in each model as assessed using the Gini impurity index, which ranks variables based on the decrease of the accuracy of an RF model when a variable is excluded, such that important variables lead to a large decrease in model performance when excluded (Breiman, 2001; Liaw & Wiener, 2002). Dashes mean a variable was not included in the final model

	Historical		Current	
	Red‐breasted	Red‐naped	Red‐breasted	Red‐naped
Maximum precipitation	1	–	4	5
Minimum precipitation	–	4	–	–
Mean annual precipitation	5	–	3	4
Precipitation seasonality	2	–	1	1
Mean temperature	6	2	7	7
Maximum temperature of warmest month	–	–	–	–
Mean maximum temperature	4	5	5	3
Mean minimum temperature	7	1	6	6
Minimum temperature of coldest month	3	3	2	2

**Table 2:** Climate variables from the MFSL (Rehfeldt et al., 2006) used in Random Forests models for both Current classifications. Number represents variable importance in each model as assessed using the Gini impurity index, which ranks variables based on the decrease of the accuracy of an RF model when a variable is excluded, such that important variables lead to a large decrease in model performance when excluded (Breiman, 2001; Liaw & Wiener, 2002). Dashes mean a variable was not included in the final model

	Current		
	Red‐breasted	Red‐naped	Hybrid
Mean temperature of coldest month	1	1	2
First freezing day of autumn	–	–	10
Mean annual precipitation	2	5	1
Growing season precipitation	–	–	4
Frost‐free period (ffp)	6	7	8
Sum of degree‐days >5°C	7	6	5
Degree‐days >5°C	–	–	7
Degree‐days <0°C	4	2	3
Min temperature of coldest month (mmin)	3	3	6
Degree‐days <0°C (based on mmin)	5	4	9

We chose three future climate scenarios from a general circulation model (GCM) based on the Canadian Center for Climate Modeling and Analysis (CGMC3). Three emission scenarios, representing low (B1), medium (A1B), and high (A2), were chosen for the year 2090 (IPCC, 2000).

### 2.3 Climate variable importance

We assessed the climate variables that were important for predicting the distributions of sapsuckers and their hybrids using Random Forests (RF) models (Breiman, 2001; Evans & Murphy, 2014; Liaw & Wiener, 2002; Cutler et al., 2007; Franklin, 2009). RF models were built using the “randomForest” (Liaw & Wiener, 2002) and “rfUtilities” packages (Evans & Murphy, 2014) as implemented in R (R Core Development Team, 2015). Models were built using species/hybrid presence as the response variable and climate variables as predictors with the following conditions: 10,000 trees (bootstrap iterations), 34% data withheld for each tree (out‐of‐bag sample), and *m* (number of independent variables permuted at each node for a tree) optimized to the out‐of‐bag error estimate (as in Evans & Cushman, 2009; Evans & Murphy, 2014; Evans et al., 2011; Murphy et al., 2010). We selected our final model using a model improvement ratio (Murphy et al., 2010), minimizing out‐of‐bag error, and maximizing parsimony (minimize the number of parameters) to maximize ecological interpretability (Evans & Murphy, 2014; Evans et al., 2011; Murphy et al., 2010). Variables used in each model were ranked by importance as assessed using the Gini impurity index. This index ranks variables based on the decrease of the accuracy of an RF model when a variable is excluded, such that important variables lead to a large decrease in model performance when excluded (Breiman, 2001; Liaw & Wiener, 2002). We visualized the relationship between important climate variables and species/hybrid presence using a nonparametric kernel density estimate (Evans et al., 2011; Simonoff, 1998). Model fit was assessed through the out‐of‐bag (OOB) error, the error rate when a given tree is predicted to the withheld (OOB) data, overall and for each class (presence vs. absence).

We conducted an independent validation of final RF models using an iterative 10% withhold (999 iterations) where 10% of the data were withheld (Evans & Cushman, 2009; Evans & Murphy, 2014; Murphy et al., 2010) to assess model performance. We measured the percent of observations that were correctly classified (PCC) and significance of the model (a nonparametric bootstrap where presence/absence is randomized and a “random” model is refit; Evans & Murphy, 2014; Murphy et al., 2010).

### 2.4 Predicted distributions

We predicted probability of occurrence across the study area based on the final model for each species and their hybrids (Evans & Cushman, 2009; Evans et al., 2011) using the “raster” package (Hijmans et al., 2015) as implemented in R (R Core Development Team, 2015). We used the PRISM climate variables to predict the historical distributions of each of the two species (PRISM Climate Group, 2013). The current predicted distributions of each of the two species were built from models using both the PRISM climate variables (PRISM Climate Group, 2013) and the variables from the MFSL (Rehfeldt et al., 2006). We predicted the current distribution of hybrid sapsuckers using only climate variables from the MFSL (Rehfeldt et al., 2006). We predicted final RF models under future climate projections to identify where currently occupied climatic conditions would exist under future climatic conditions (Evans & Cushman, 2009). We predicted the distribution of each species and their hybrids under three different emission scenarios using a GCM from the CGMC3: low (B1), medium (A1B), and high (A2) emission scenarios for the year 2090 (Rehfeldt et al., 2006).

We were unable to assess the full extent of overlap, and thus the area of the hybrid zone, between the parental species because our models of species distribution were weakest along the edges of their distribution in the contact zone. To model the distribution of the hybrid zone, we constructed distribution models based on localities where birds with hybrid phenotypes were observed. We assumed that localities where hybrid were observed represented areas within the contact zone, as hybrids typically do not occur outside of the region of the contact zone during June and July (Howell, 1952; Johnson & Johnson, 1985). We used localities where sapsuckers with hybrid phenotype were observed to predict the distribution of the hybrid zone. We had a sufficient sample size of localities where sapsuckers with hybrid phenotypes were observed to construct a hybrid phenotype distribution model under current climate conditions (1972–2013). To maintain a balanced model and meet model assumptions (Evans & Cushman, 2009; Evans et al., 2011), we used a random subset of the absences (*n *=* *300) relative to current climate models for each parental species. Using the RF model of hybrid occurrence, we predicted the distribution hybrids across the landscape. To determine the effect of climate change on the future of where hybrid individuals are found in the region, and thus where the hybrid zone occurs, we also projected the distribution of hybrids into the future using the same three future climate scenarios (B1, A1B and A2) used to predict the distribution of Red‐naped and Red‐breasted Sapsuckers.

Given the difficulty of identifying hybrids, we also ran models where we randomly added 10% and 20% simulated error into our hybrid observations to assess how robust our models were to potential identification error. We introduced error in two ways: (1) we randomly removed observations of hybrids, reducing our sample size and simulating misidentification of birds with hybrid phenotypes as parental species and (2) we randomly assigned hybrid identity to parental observations, simulating misidentification of parental species as hybrids. We weighted our selection of parental observations based on the probability they were found within the predicted distribution of the hybrid zone (based on the predicted distribution of individuals with hybrid phenotypes from the unaltered dataset).

We tested for significant differences between historical and current distributions for both Red‐naped and Red‐breasted sapsuckers. We directly compared these distributions to determine areas of significant range increases and decreases for each species. Significance was calculated by assessing the pairwise differences of the two distributions relative to the mean and variance of differences (R package SDMTools: VanDerWal, Falconi, Januchowski, Shoo, & Storlie, 2014).

### 2.5 Climate niche similarity

To account for differences in the types of locality data we used between our historical and current models (Tingley & Beissinger, 2009), we reran our current models using only a subset of the locality data. To directly compare between historical models, which used only museum specimen records, and current models, we built current models using only museum records (*n *=* *99). We used the same climate variables from the PRISM Climate Group (2013) for both historical and current models that used only museum records. We used these current models constructed with museum records to directly compare occupied climate niche space between historical and current periods to avoid the potential for differences in locality data driving our results (see Tingley & Beissinger, 2009).

We tested the effect of climate on the shifts of distributions of Red‐naped and Red‐breasted sapsuckers between historical and current models by directly comparing the climate niche space occupied between the two periods using two approaches. First, using models built only with museum records, we calculated the hypervolume space each climate niche occupied by modeling the first three axes of the multidimensional space of each species using historical and current data. Using the “hypervolume” package (Blonder, 2015) as implemented in R (R Core Development Team, 2015), we calculated the overlap between historical and current climate niche hypervolume spaces for both Red‐naped and Red‐breasted sapsuckers. We used the Sörensen similarity index (Hanson, 1955; Sörensen, 1948) as implemented in the “hypervolume” package (Blonder, 2015), which ranges from 0 (completely different) to 1 (identical) to quantify niche space overlap. As a separate test of climate niche similarity between historical and current periods, we also used the current predicted niche and reprojected the distribution of that niche space under historical climate conditions (Evans & Cushman, 2009; Evans et al., 2011) using the raster package (Hijmans et al., 2015). This allowed us to directly compare the distributions of the occupied niches and to determine how climate change alone impacted the niche and distributions of each sapsucker species.

## 3 Results

### 3.1 Climate niche and variable importance

**Figure 3:**
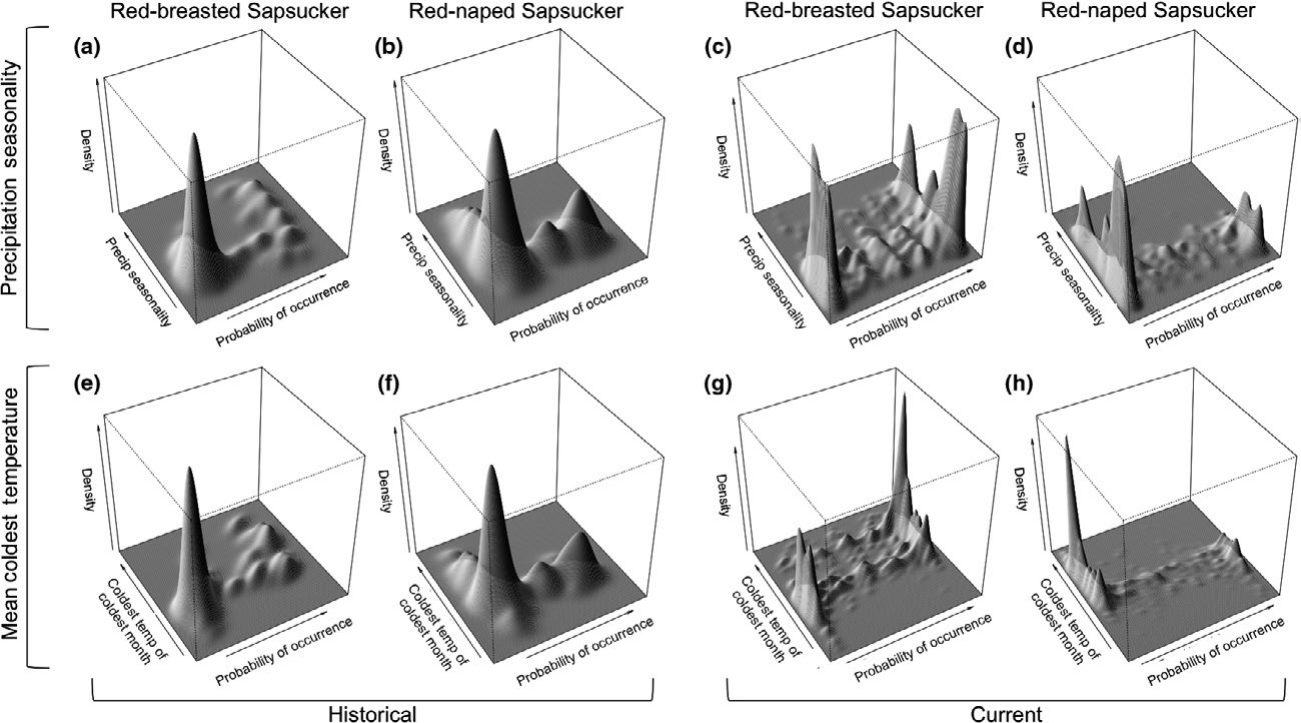
Bivariate kernel density plots of two climate variables using climate variables from the PRISM Climate Group (2013). Bivariate kernel density plots of precipitation seasonality shown in top row (plots a–d), while the bivariate kernel density plots for coldest temperature of the coldest month (January minimum temperature) are shown in the bottom row (plots e–h). Plots from historical models (a, b, e, f) and current models (c, d, g, h) for Red‐breasted (a, c, e, g) and Red‐naped sapsuckers (b, d, f, h) are shown. Plots show over what range of values sapsuckers are likely either to be present or absent. Peaks represent how likely each species are to be present (high probability of occurrence) or how likely a species are to be absent (low probability of occurrence) over a range of values for a given climate variable

We were able assess which climate variables (Table 1) were important for parental sapsuckers using past climate conditions and museum specimen localities. While all models were significant (*p*‐values .0347–.0001), the Red‐breasted Sapsucker historical model received higher support than the Red‐naped Sapsucker model, both in terms of fit (OOB: 12.98% and 27.48%, respectively) and validation (PCC: 80.20% and 55.58%, respectively; Table 3). The final Red‐breasted Sapsucker historical model of species occurrence (see 2 for model selection) contained seven selected variables; maximum precipitation, precipitation seasonality, and minimum temperature of the coldest month were the top three variables important in predicting the niche of Red‐breasted Sapsuckers (Table 1). The final Red‐naped Sapsucker historical model of species occurrence contained five selected variables; mean minimum temperature, mean annual temperature, and minimum temperature of the coldest month were the three most important variables (Table 1). Red‐breasted Sapsucker historical probability of occurrence was associated with moderate values for coldest temperatures, moderate‐to‐high values for mean annual temperature, and a relatively wide range of precipitation seasonality values, while probability of absence was associated with low extremes of these three variables based on bivariate kernel density plots (Figure 3). The probability of occurrence of Red‐naped sapsuckers was associated with lower cold temperatures than Red‐breasted Sapsuckers, moderate mean annual temperatures, and low‐to‐moderate precipitation seasonality values. Red‐naped Sapsucker probability of absence, however, was associated with a broad range of mean annual temperature values and cold temperatures (Figure 3).

**Table 3:** Random Forests model statistics for current models of Red‐breasted, Red‐naped, and hybrid sapsucker climate niches, and for historical models of Red‐breasted and Red‐naped sapsucker climate niches. Model statistics from models using climate variables from the PRISM Climate Group (2013) are presented for both current and historical models of Red‐breasted and Red‐naped sapsuckers. Model statistics from the model using climate variables from the MFSL (Rehfeldt et al., 2006) are presented for the model of hybrid phenotypes. Out‐of‐bag error (OOB) is given as: overall error (error in presence class, error in absence class). Percent correctly classified (PCC) is given as overall error. The *p*‐values for each model, as assessed using a nonparametric bootstrap method (Evans & Murphy,^2014^; Murphy et al., 2010), are also given

	Out‐of‐bag error	PCC	*p*‐Value
Current models			
Red‐breasted	15.70 (11.03, 21.85)	83.57	.001
Red‐naped	11.25 (21.50, 7.06)	86.32	.001
Hybrid	17.19 (41.59, 8.00)	73.00	.001
Historical models			
Red‐breasted	12.98 (28.95, 6.45)	80.20	.0001
Red‐naped	27.48 (63.64, 15.31)	55.58	.0347

Models of current distribution were well supported for both parental species. Using climate variables from the PRISM Climate Group (2013), model fit was high for both species (OOB 15.70% Red‐breasted, 11.29% Red‐naped; Table 3). Model validation statistics, including percent correctly classified, were moderate for both Red‐breasted and Red‐naped Sapsucker models (PCC values of 83.57% and 86.32%, respectively; Table 3). Both Red‐naped and Red‐breasted current models were highly significant (*p*‐value .001; Table 3). Seven variables were retained after model selection in the final Red‐breasted Sapsucker model, with precipitation seasonality explaining the most variation in occurrences, nearly twice as much as the next most important variable (minimum temperature of coldest month; Table 1). Seven variables were also retained in the final Red‐naped Sapsucker model, with precipitation seasonality again coming out as the most important variable, explaining the greatest amount of variation in the model (Table 1). Bivariate kernel density plots of two important climate variables for each species show differences in occupied climate space (Figure 3). Red‐breasted Sapsucker probability of occurrence was associated with a wide range of precipitation seasonality variables, ranging from low to high, while there was a high probability of being absent from areas that experience only low values of precipitation seasonality (Figure 3). A probability of occurrence of Red‐naped Sapsuckers, on the other hand, was associated with low precipitation seasonality, with a high probability of absence in areas that experience a wide range of values, from low‐to‐high precipitation seasonality (Figure 3). Red‐breasted Sapsucker probability of occurrence was also strongly associated with moderate‐to‐warm winter temperatures as measured by the mean coldest temperature of the coldest month, but were predicted to show a high probability of absence from areas that experience the coldest winter temperatures (Figure 3). Conversely, Red‐naped Sapsucker probability of occurrence was associated with cold winter temperatures, while a high probability of absence was associated with areas that experience moderate‐to‐warm winter temperatures (Figure 3).

**Figure 4:**
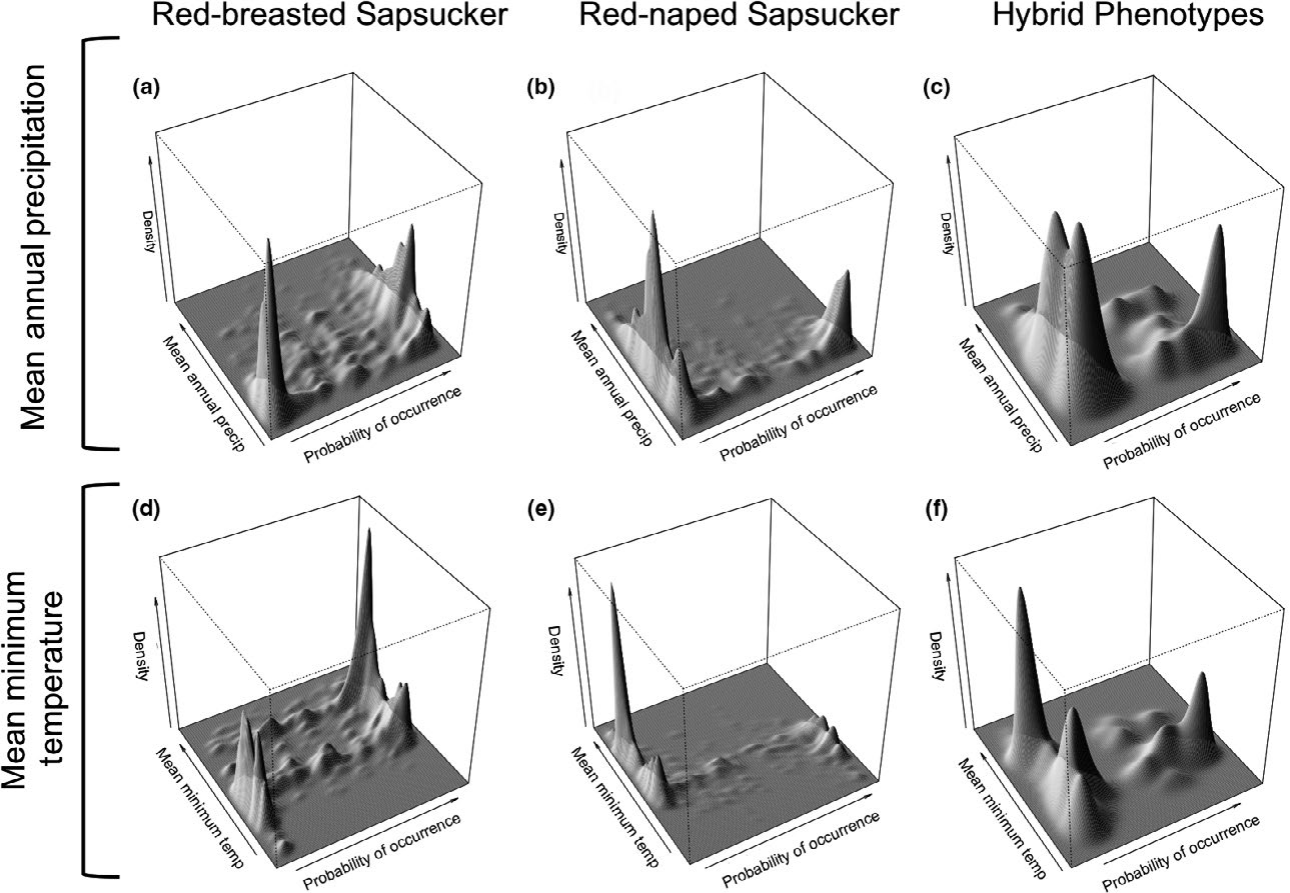
Bivariate kernel density plots of two climate variables of importance from current Random Forests models for Red‐breasted (plots a and d), Red‐naped (plots b and e), and hybrid sapsuckers (plots c and f). Plots show over what range of values sapsuckers are likely either to be present or absent. The climate variables modeled here come from the MFSL (Rehfeldt et al., 2006). Peaks represent how likely each species are to be present (high probability of occurrence) or how likely a species are to be absent (low probability of occurrence) over a range of values for a given climate variable. Plots a–c are bivariate kernel density plots for mean annual precipitation; plots d–f are plots for mean minimum temperature

To assess the influence of climate data on results, we compared models of current distribution based on MFSL (Rehfeldt et al., 2006) data to our results using climate variables from the PRISM Climate Group (2013). Current RF models based on data from the MFSL (Rehfeldt et al., 2006) were comparable to those using data from the PRISM Climate Group (2013). Models were well supported for both species: model fit was high for both species (OOB 15.7% Red‐breasted, 21.85% Red‐naped; Table S4), and both models were highly significant (*p*‐value .001; Table S4). As in the models built using climate variables from the PRISM Climate Group (2013), winter temperatures were also important predictors of the climate niche of both species using climate variables from the MFSL (Rehfeldt et al., 2006; Table 2). Red‐breasted Sapsucker probability of occurrence was associated with moderate‐to‐warm winter temperatures, while there was a high probability of absence associated with colder winter temperatures using data from the MFSL (Rehfeldt et al., 2006; Figure 4). A probability of occurrence of Red‐naped Sapsuckers, on the other hand, was associated with colder winter temperatures, with a high probability of absence associated with areas that experience warmer winter temperatures (Figure 4). Red‐breasted Sapsucker probability of occurrence was also strongly associated with a wide range of moderate mean annual precipitation values, while a high probability of absence is associated with very dry conditions as measured by mean annual precipitation. Unlike Red‐breasted Sapsuckers, Red‐naped Sapsucker probability of occurrence was associated with low mean annual precipitation, while a high probability of absence was associated with areas that experience a range of moderate mean annual precipitation (Figure 4).

Hybrid zone RF models based on the localities of individuals with hybrid phenotypes were not as strongly supported as those for parental species. Overall model fit was moderate (OOB 17.19%). Model validation statistics, including PCC, were also moderate (72.75%; Table 3). The hybrid model was significant (*p*‐value .001; Table 3). We retained ten variables for the final hybrid model, with mean annual precipitation explaining the most variation (Table 2). Bivariate kernel density plots of the top two most important climate variables show hybrid individuals have a high probability of occurrence associated with moderate winter temperatures, while having a high probability of absence associated with areas that experience both colder and warmer winter temperatures (Figure 4). Hybrid probability of occurrence was also associated with low mean annual precipitation, while they showed a high probability of absence associated with areas that experience a range of moderate precipitation levels (Figure 4).

### 3.2 Predicted distributions

**Figure 5:**
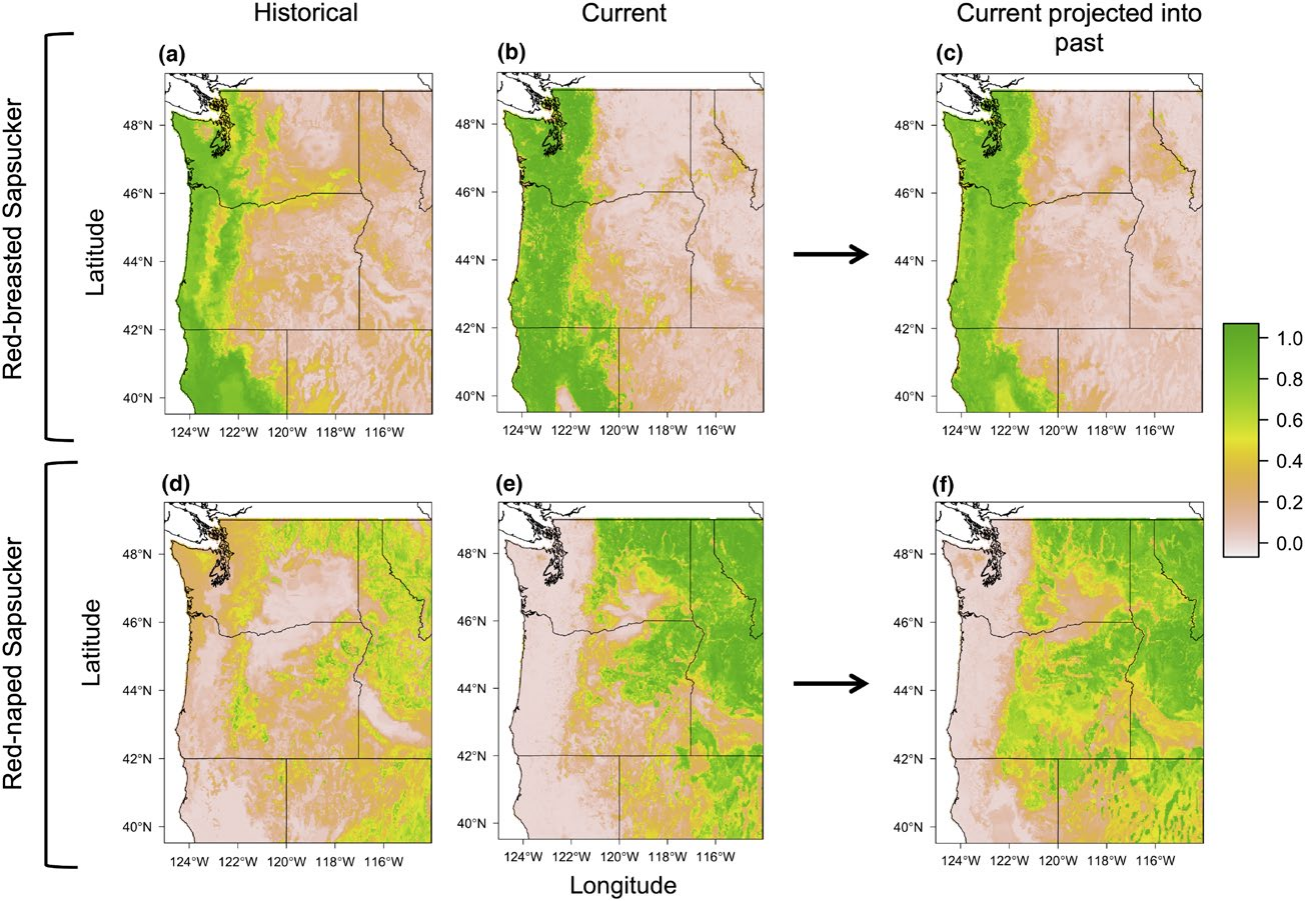
Predicted distributions for Red‐breasted and Red‐naped sapsuckers under historical climate conditions using historical locality data (1910–1940; plots a and d) and current climate conditions using current locality data (1970–2000; plots b and e). We used data from the PRISM Climate Group (2013) to predict the historical and current distribution of sapsuckers in these plots. Plots c and f represent the distribution of the current climate niche generated using current locality data projected into the past (1910–1940) using historical climate data to show where the current climate niche existed under historical climate conditions. Plots a–c represent predicted distributions for Red‐breasted Sapsucker; plots d–f represent predicted distributions for Red‐naped Sapsucker. Color scale represents probability of presence ranging from 0 to 1, with pale pink representing a probability of occurrence of 0, and green representing a probability of occurrence of one

Historical models show each species occupied distinct climate niches, which generally matched what was known about the distributions of both species (Howell, 1952; Johnson & Johnson, 1985; Figure 5a,d). Red‐breasted Sapsuckers were completely absent from much of eastern California in the historical predicted distribution. Conversely, the predicted historical distribution of Red‐naped Sapsucker is patchily distributed across the landscape, with distributions mainly restricted to mountainous regions throughout the interior west, east of the Cascade Range.

**Figure 6:**
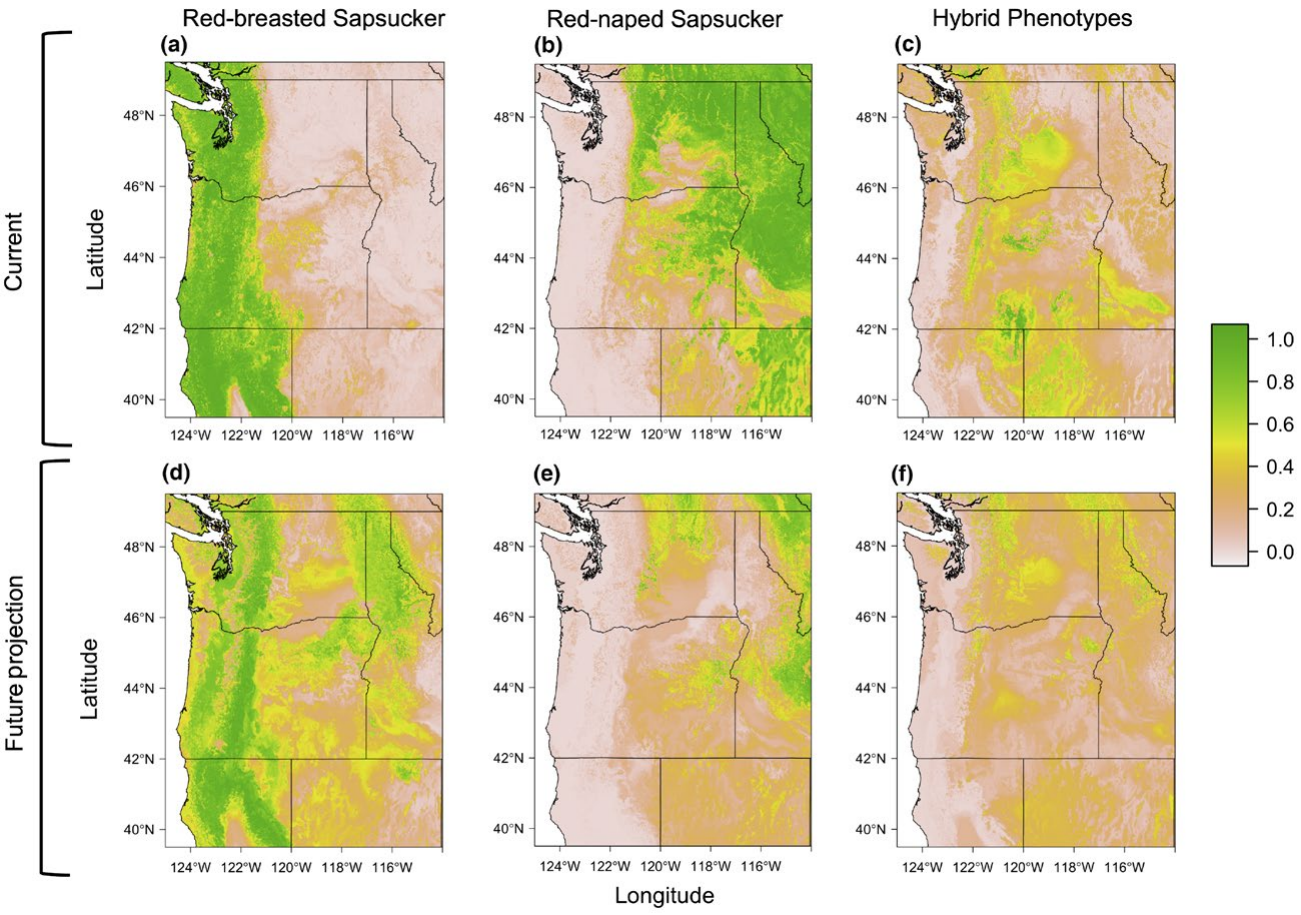
Predicted distribution for Red‐breasted, Red‐naped, and hybrid sapsuckers under current (1970–2000; plots a through c) and future climate conditions (A1B scenario: 2090; plots d through f). We used data from the MFSL (Rehfeldt et al., 2006) to predict the current and future distributions of the climate niche of sapsuckers in these plots. Plots a and d show the current and future distributions of Red‐breasted Sapsuckers; plots b and e, the current and future distributions of Red‐naped Sapsuckers; and plots c and f, the current and future distributions of hybrid phenotypes. The predicted distribution of sapsuckers with hybrid phenotypes is assumed to correspond to the hybrid zone as hybrids are only encountered within the hybrid zone during the survey period (Howell, 1952; Johnson & Johnson, 1985; Trombino, 1998). Color scale represents the probability of presence ranging from 0 to 1, with pale pink representing a probability of occurrence of 0, and green representing a probability of occurrence of one

Predicted current distributions for both Red‐naped and Red‐breasted sapsuckers using both PRISM climate data and data from the MFSL (Rehfeldt et al., 2006) closely match what is known for each species, both based on current literature (Walters et al., 2014a,b) and personal observations within the hybrid zone (Figures 5b,e and 6a,b). Red‐breasted Sapsuckers showed significant range expansion into northeast California and south‐central Oregon from the historical period to the current period, while Red‐naped Sapsuckers showed significant range loss from this same region (Figures 5 and 6). Differences in Red‐naped Sapsucker distributions were more difficult to detect between historical and current models, as historical predicted distribution models were so patchy and not well supported.

Predictions for the ranges of both parental species for all future climate scenarios showed drastic shifts in spatial location of currently occupied climate conditions that could indicate a shift in distributions. Red‐breasted Sapsuckers showed range expansion to the east of their current range and range retraction near the Pacific coast and higher elevations of the Cascades under all tested future climate change scenarios (Figure 6a,d), while Red‐naped Sapsuckers showed consistent range contraction, seeming to retract to higher elevations from where they are currently distributed (Figure 6b,e). The amount of predicted future distribution change followed predictably with the amount of climate change predicted in each future scenario: the lowest emission scenario saw the least predicted range change, while the highest emission scenario saw the greatest change in the predicted distribution.

The predicted distribution of hybrid phenotypes closely matched the known distribution of the hybrid zone east of the Cascade Range, through northeast California, and into central and southeastern Oregon (Johnson & Johnson, 1985; Trombino, 1998). In all future climate scenarios, hybrid distributions were greatly reduced, with only a low probability of hybridization along some mountain ranges in Oregon, Washington, and Idaho (Figure 6c,f). We found that models with simulated random error were consistent with our original, unaltered dataset of hybrid observations. This suggests that our hybrid models are robust even in the face of some identification error, giving us additional confidence in the power of our models (see Supplemental Information, Figure S3).

### 3.3 Climate niche similarity

Current climate models built using only museum records and PRISM data were similar to those modeled using the full dataset of current sapsucker observations (Figures 5 and S4). There were differences, however, between the two sets of distributions, with the current predicted distribution using only museum records showing gaps and lack of detail from climate space where sapsuckers were not sampled (Figure S4). The predicted current distribution using the full locality dataset shows a broader distribution, especially for Red‐breasted Sapsuckers (Figure 5). However, both current predicted distributions show the same range expansion of Red‐breasted Sapsuckers into northeast California and central Oregon, where they were not predicted to occur using historical models.

We tested the similarity of the climate niche occupied by Red‐naped and Red‐breasted sapsuckers under historical and current climate conditions using the Sörensen similarity index (Blonder, 2015; Hanson, 1955; Sörensen, 1948). We found that the historical and current occupied climate niches were similar for both Red‐naped and Red‐breasted sapsuckers. We found that historical and current Red‐naped Sapsucker distribution models had a Sörensen similarity index value of 0.7, while historical and current Red‐breasted Sapsucker distribution models had a Sörensen similarity index value of 0.72, respectively. We also determined where the currently occupied climate niche would have existed on the landscape under historical climate conditions. We found that, especially for Red‐breasted Sapsuckers, the projected distribution of the current climate niche under historical climate conditions matched very closely to the historical distributions of each species (Figure 5). The distribution of the Red‐naped Sapsucker current climate niche did not match well with the historical predicted distribution because of the weak performance of the historical model (Table 3).

## 4 Discussion

We sought to determine the climatic niche of two species of sapsucker and their hybrids in the Pacific Northwest of the United States to better understand the role climate plays in structuring a rapidly moving hybrid zone. Using these climatic niches, we were able to accurately predict historical and current distributions for both Red‐naped and Red‐breasted sapsuckers (Figures 5a,b,d,e and 6a,b) and the extent of the hybrid zone based on hybrid locality data (Figure 6e). Aspects of precipitation regime, especially precipitation seasonality, and cold winter temperatures, both in terms of duration and degree, were particularly important in predicting where parental sapsuckers would occur. Red‐breasted were predicted to be in areas with warmer, shorter winters, as well as a wide precipitation gradient, being absent from only the most seasonal, dry conditions. Red‐naped Sapsuckers, on the other hand, were predicted to occur in areas with colder temperatures, longer winters, and areas that experienced drier conditions and very seasonal precipitation patterns. Like the parental species, winter temperature was important in predicting where the hybrid phenotypes, and thus the hybrid zone, occurs, in addition to the amount of precipitation over the year (Table 2, Figure 4). Further, we demonstrated that climate change is associated with range shifts of parental species over the past 100 years and is predicted to impact where these two species come into contact in the future.

Despite their ecological similarities, Red‐naped and Red‐breasted sapsuckers occupied distinctly different climate niche space. While the occupied climate space for each species was different, many of the same variables were important for each species. For example, precipitation seasonality and minimum temperature of the coldest month were the two most important predictors of occurrence, explaining the most variation in occurrence for each species (Table 1). Using data from MFSL (Rehfeldt et al., 2006), three additional top‐ranked variables were related to cold temperatures, with Red‐breasted Sapsuckers consistently found in areas experiencing shorter, warmer winter conditions and Red‐naped Sapsuckers conversely found in areas with longer, colder winter conditions (Table 2). The climate niche occupied by hybrids where the hybrid zone occurs shared characteristics with the climate niches of both parental species, but did not match either one exactly. While the hybrid zone is associated with areas that experience low mean annual precipitation, similar to Red‐naped Sapsuckers, the hybrid zone is also characterized by moderate winter temperatures, similar to Red‐breasted Sapsuckers. This emphasizes the important role climate change may have in structuring the distribution of these species and the hybrid zone between them.

Models of the historical distributions of sapsuckers generally received lower support than current models, likely a result of many fewer sampling localities. While current models for each species generally performed very well, predictions were weakest at the edges of their distributions, where the two species would come into contact and hybridize. Weak model performance at the edges of distributions could be the result of each species reaching the edge of its ecological tolerance, or more simply a result of a smaller sample size, leading to a lower signal‐to‐noise ratio (Austin, 2002). Though not as strongly supported as the distribution of the parental species, likely a result of a small sample size, the predicted distribution of hybrid phenotypes nonetheless matched the current extent of the hybrid zone in the Pacific Northwest.

Just as climate is an important factor in shaping the distributions of species and patterns of hybridization, a changing climate has the ability to alter how species interact and thus their evolutionary trajectories by changing the degree to which taxa may overlap and hybridize (e.g., Garroway et al., 2010, 2011). Sapsucker distributions have already shifted significantly, especially in Oregon and parts of California. We show that, by comparing predicted distributions of the two parental species on the basis of climate variables, shifts in the distribution of sapsuckers is likely influenced by shifts in the climate niche space of each species. Using a measure of niche space and projecting the currently occupied climate niche of sapsuckers into the past using historical climate conditions, we show that these changes in distribution between historical and current periods are the result of sapsuckers tracking a changing climate (Figure 5). The climate niche space occupied by sapsuckers is similar between historical and current periods, suggesting that observed shifts in the distributions of each species are the result of tracking the same niche as it moves across the landscape in response to climate change, as opposed to other intrinsic mechanisms of hybrid zone movement. Tingley, Monahan, Beissinger, and Moritz (2009) also showed support for birds in California tracking their Grinnellian climate niche in response to climate change over a similar time period.

In addition to documenting the shifts in the distributions of sapsuckers under past and current conditions, we also used the current climate envelope of each sapsucker to predict that observed range shifts may continue into the future, as the climate space occupied by each species is predicted to shift drastically. While Red‐breasted Sapsuckers are predicted to expand their range greatly into the future, Red‐naped Sapsuckers are predicted to experience range retraction, seemingly becoming isolated in higher elevations (Figure 6c,d). Based on field observations, we initially predicted that Red‐breasted Sapsuckers were expanding into and replacing Red‐naped Sapsuckers (Rhymer & Simberloff, 1996), possibly the result of patterns of differential aggression (Billerman & Carling, in review) or mate choice (Johnson & Johnson, 1985). Based on their different climate niches, however, it instead appears that Red‐breasted Sapsuckers are expanding eastward as habitat becomes suitable, while simultaneously, the same habitat is becoming unsuitable for Red‐naped Sapsuckers.

Like the predicted distributions for each parental species, the predicted distribution and extent of the hybrid zone, based on hybrid phenotypes, will change drastically in the future (Figure 6f). The climate envelope that currently defines the hybrid zone where the parental species overlap is predicted to shrink substantially and in some future climate scenarios disappear entirely. This suggests that future climate change may lead to less range overlap between the parental species, and therefore less potential for hybridization. Our prediction that climate may contribute to a reduction and possibly even cessation of hybridization is unusual; in all known cases where climate has lead or is likely to lead to movement in a hybrid zone, levels of hybridization are either maintained (Taylor et al., 2014; Walls, 2009), or hybridization increases due to increased range overlap (e.g., Garroway et al., 2010, 2011) or due to changes in selection pressures on hybrids (e.g., Engebretsen et al., 2016). This pattern may reflect a novel climate niche existing now that may disappear in the near future, possibly leading to different species associations. A novel habitat niche has possibly contributed to the expansion and success of hybrids in a chorus frog hybrid zone and leading to a potential breakdown of reproductive isolation (Engebretsen et al., 2016). Such novel climate niches with unique species associations have also been demonstrated in paleoclimate studies where no analog plant and climate systems existed (Williams & Jackson, 2007) and are predicted to develop with future climate change (Williams, Jackson, & Kutzbach, 2007).

While many studies of hybridization focus on understanding the mechanisms that contribute to intrinsic reproductive isolation (Coyne & Orr, 2004; Harrison, 1993), prezygotic isolating barriers, such as niche or habitat differences, are often much more important reproductive barriers (Schemske, 2010; Sobel, Chen, Watt, & Schemske, 2010). We argue that our findings of climate niche differences in sapsuckers conforms to the concept of ecogeographic isolation (Schemske, 2010), wherein largely nonoverlapping ranges, which are due to adaptive differentiation to different habitats or climate niches, result in a reduction of gene flow between populations. In *Mimulus*, for example, geographic differences and associated differences in occupied niche are heritable and are very important to reproductive isolation (Schemske, 2010; Sobel et al., 2010). Our models predicting a reduction of hybridization in the future certainly suggests that tracking current climate niches will lead to further isolation of sapsuckers, perhaps reinforcing current barriers (Funk, Nosil, & Etges, 2006). While we cannot know how the differences in climate niche of sapsuckers will ultimately contribute to further reproductive isolation, such adaptive differences to ecological conditions can result in intrinsic reproductive isolation incidentally as a result of natural selection on morphological, behavioral, or physiological traits (Funk et al., 2006; Schemske 2010, Schluter, 2001; Sobel et al., 2010).

To account for differences in locality data used between historical and current models, we built current models using only locality data from museum records. We do not see a strong influence of data type on our results, as has been demonstrated in other studies (e.g., Tingley & Beissinger, 2009), with different sets of climate variables (PRISM vs. MFSL) and locality data (museum records vs. eBird) recovering the same overall effects of climate on sapsuckers in the northwestern United States. Using only museum records, we found a similar pattern of range expansion from the historical to the current period, reinforcing our conclusions of change that is not just the result of different qualities and quantities of locality data.

While climate change may be directly impacting these distribution changes in sapsuckers through physiological intolerances or competitive exclusion, these shifts may also be the result of indirect associations that impact aspects of habitat (Thuiller et al., 2008). Though ecologically very similar, each species occupies different forest types. Red‐breasted Sapsuckers are able to take advantage of a wide range of forest types, while Red‐naped are more restricted, and are often strongly tied to quaking aspen (*Populus tremuloides*) groves for nest sites (Crockett & Hadow, 1975; Walters et al., 2014a,b). In western North America, climate change is predicted to have profound effects on the structure of many of the forest habitats occupied by one or both species of sapsucker (Mantgem et al., 2008; Rehfeldt et al., 2006, 2009; Worrall et al., 2013). Quaking aspen is predicted to be especially susceptible to climate change and has already shown drastic declines through much of western North America, especially in Oregon and California (Anderegg et al., 2012; Hanna & Kulakowski, 2012; Rehfeldt et al., 2009). These declines have been linked to rising temperatures and drought stress (Anderegg et al., 2012; Hanna & Kulakowski, 2012; Rehfeldt et al., 2009). A study investigating the impact of future climate change on the distribution of aspen show similar patterns to those seen in Red‐naped Sapsuckers, with aspen distribution continuing to retract and becoming isolated at higher elevations (Rehfeldt et al., 2009). Declines in aspen are unable to describe the simultaneous expansion of Red‐breasted Sapsucker, but the potential decline of Red‐naped Sapsuckers with aspen disappearance may allow for easier colonization of these now vacant habitats by Red‐breasted Sapsuckers.

Shorter and warmer winters may contribute to sapsucker distribution shifts in several ways. Migration distance and timing likely both play an important role, as Red‐breasted Sapsuckers generally migrate shorter distances to wintering grounds and arrive earlier to breeding grounds than Red‐naped Sapsuckers (Trombino, 1998; Walters et al., 2014a,b). Red‐breasted Sapsuckers may be able to compensate for changing conditions more effectively than long‐distance migrants (Both, Bijlsma, & Visser, 2005; Both et al., 2009). Further, in many birds, individuals that manage to establish territories experience low turnover, regardless of competitive ability (Chalfoun & Schmidt, 2012). As Red‐breasted Sapsuckers arrive earlier than Red‐naped Sapsuckers (Trombino, 1998), they are able to obtain territories before Red‐naped Sapsuckers. As a result, Red‐naped sapsuckers may be unable to acquire territories, pushing them out of the area. In addition to territory establishment, changes in the timing of winter and the subsequent onset of spring can influence the timing of peak insect abundance, which is extremely important for breeding success in many species (Thomas et al., 2001). Even if neither species is able to significantly adjust their migration timing, Red‐breasted Sapsuckers would be best suited to take advantage of the earlier peak insect abundance that would occur with an earlier onset of spring, while Red‐naped Sapsuckers may experience population declines. Red‐breasted Sapsuckers may thus expand into these areas that were once occupied by Red‐naped Sapsuckers in the wake of their decline. Steep population declines have been observed in many other avian systems as a result of phenological mismatch, when species or populations are unable to match the pace of climate change and thus the peak of food abundance (Saino et al., 2011; Thomas et al., 2001).

Adaptation to new and changing environmental conditions in the future can alter the relationships that species currently have with their environment, greatly changing the predictability of future range change (Davis & Shaw, 2001; Davis, Shaw, & Etterson, 2005). While adaptation is certainly a possibility in our system, we feel it may not have a strong impact on our ability to predict the future distribution, as the climate niche of both Red‐naped and Red‐breasted sapsuckers is very similar across historical and current models, suggesting that both species are tracking the same or similar climate niche through time. Further, we also show that the current climate niche predicts very well the historical distribution, providing additional evidence that the climate niche between these two periods has not changed for either species. However, if Red‐naped Sapsuckers are indeed able to adapt to new changing environmental conditions, this may lead to a scenario of increased hybridization, as current predictions suggest that range overlap and hybridization will decrease if species stay restricted to their current ecological and climate niche.

Climate has still not been widely demonstrated to be the cause of many moving hybrid zones (but see Carling & Zuckerberg, 2011; Garroway et al., 2010, 2011; Taylor et al., 2014, 2015; Walls, 2009) with only anecdotal evidence in many cases (Buggs, 2007). We contribute to the growing collection of studies that demonstrate the effect of climate on hybridizing taxa. In the sapsucker hybrid zone system, precipitation regime and warmer winter temperatures were both found to be extremely important factors in the eastward expansion of Red‐breasted Sapsuckers and the simultaneous retraction of Red‐naped Sapsuckers, greatly altering where these two species may come into contact and hybridize. The distributions of sapsuckers are predicted to continue to shift with projected future climate change, potentially resulting in less range overlap and lower rates of hybridization. We have shown that exogenous factors, such as climate, help shape the evolutionary trajectories of speciation events.

## Conflict of Interest

None declared.
