# Supplementary figures and images for: Changing climate mediates sapsucker (Aves: Sphyrapicus) hybrid zone movement
Source: Ecol Evol. 2016 Oct 12;6(22):7976–90. doi: 10.1002/ece3.2507 (PMC5108250; doi:10.1002/ece3.2507)

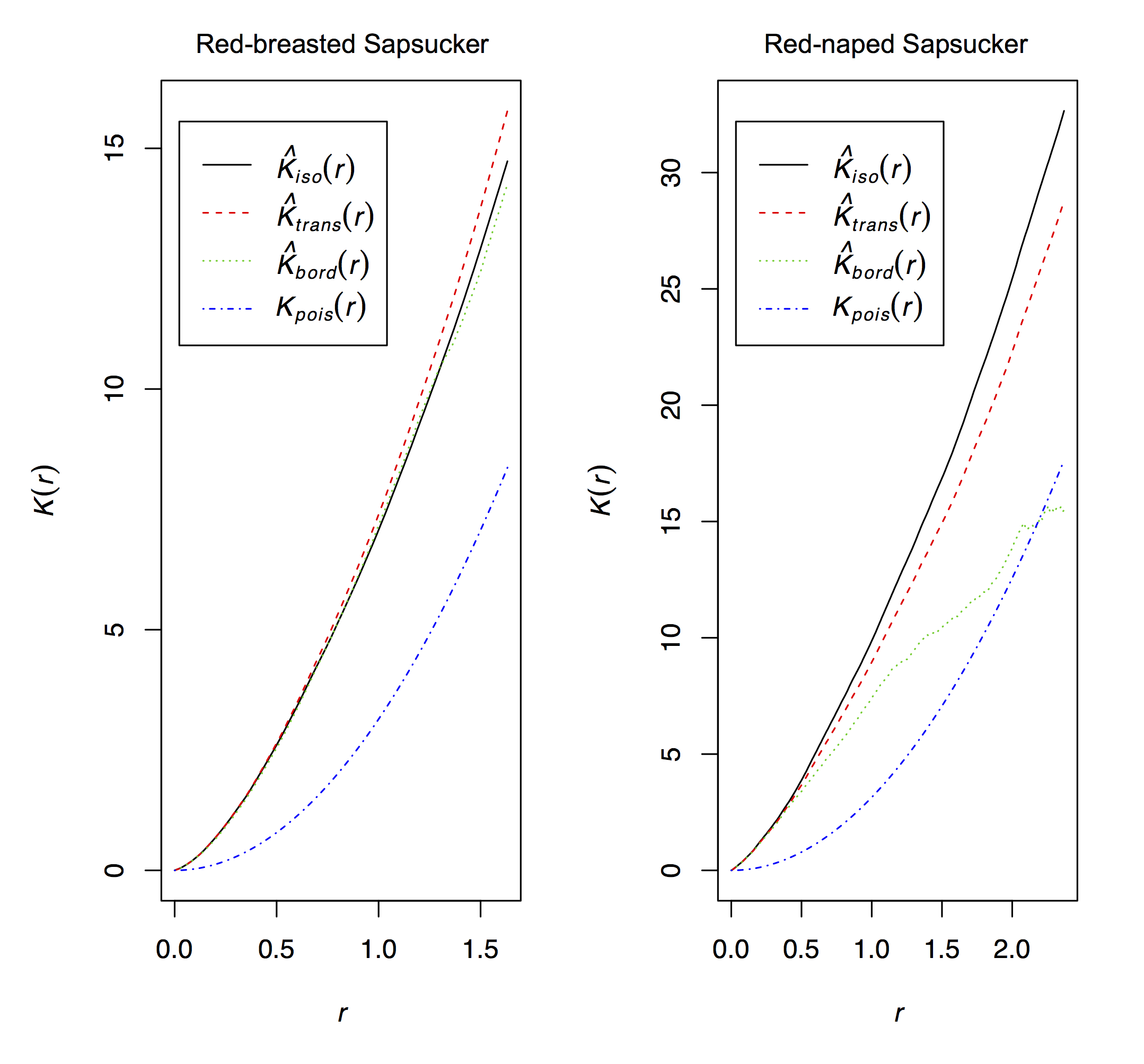

Supplement: Supplementary file 1 [file ECE3-6-7976-s001.tiff]

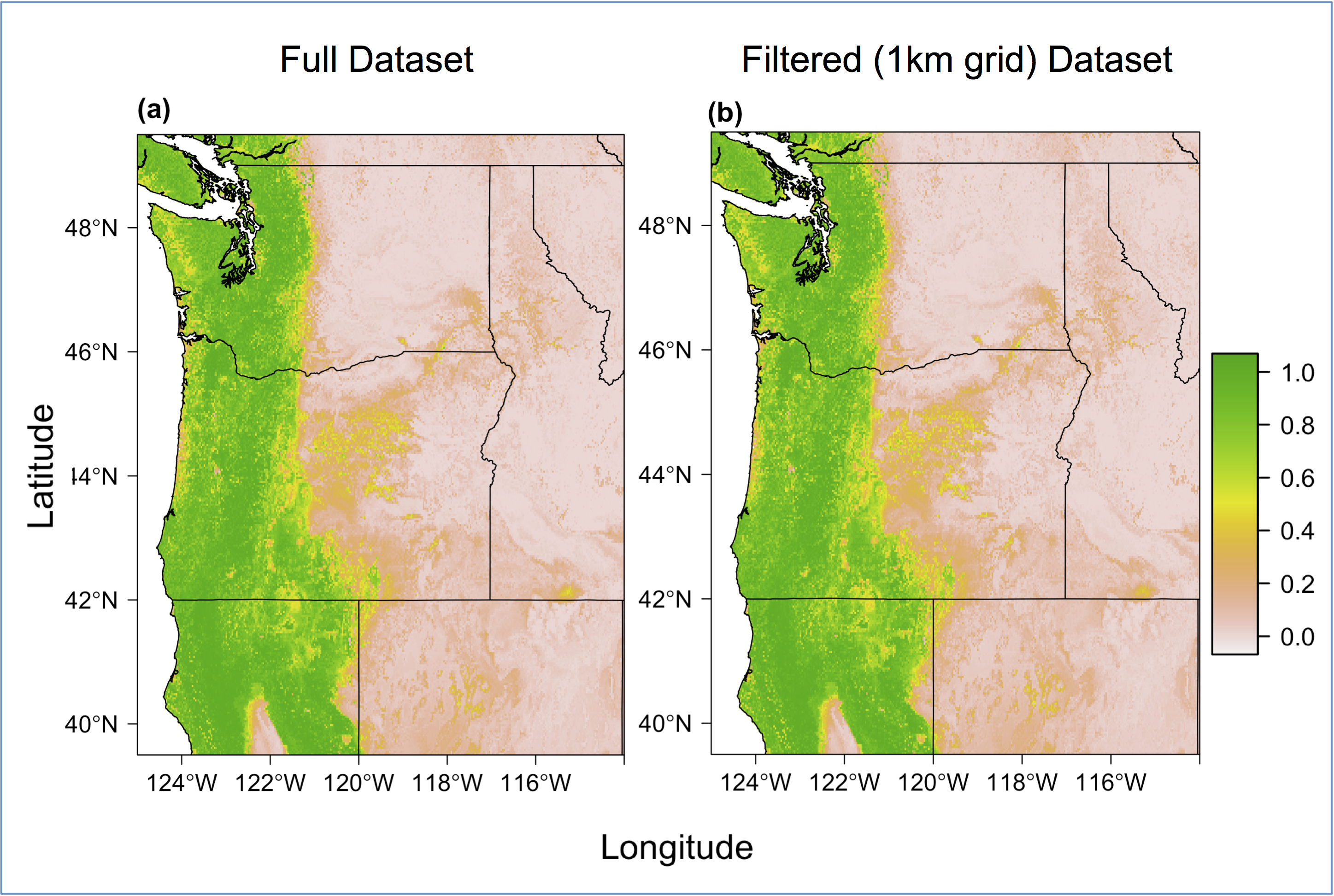

Supplement: Supplementary file 2 [file ECE3-6-7976-s002.tiff]

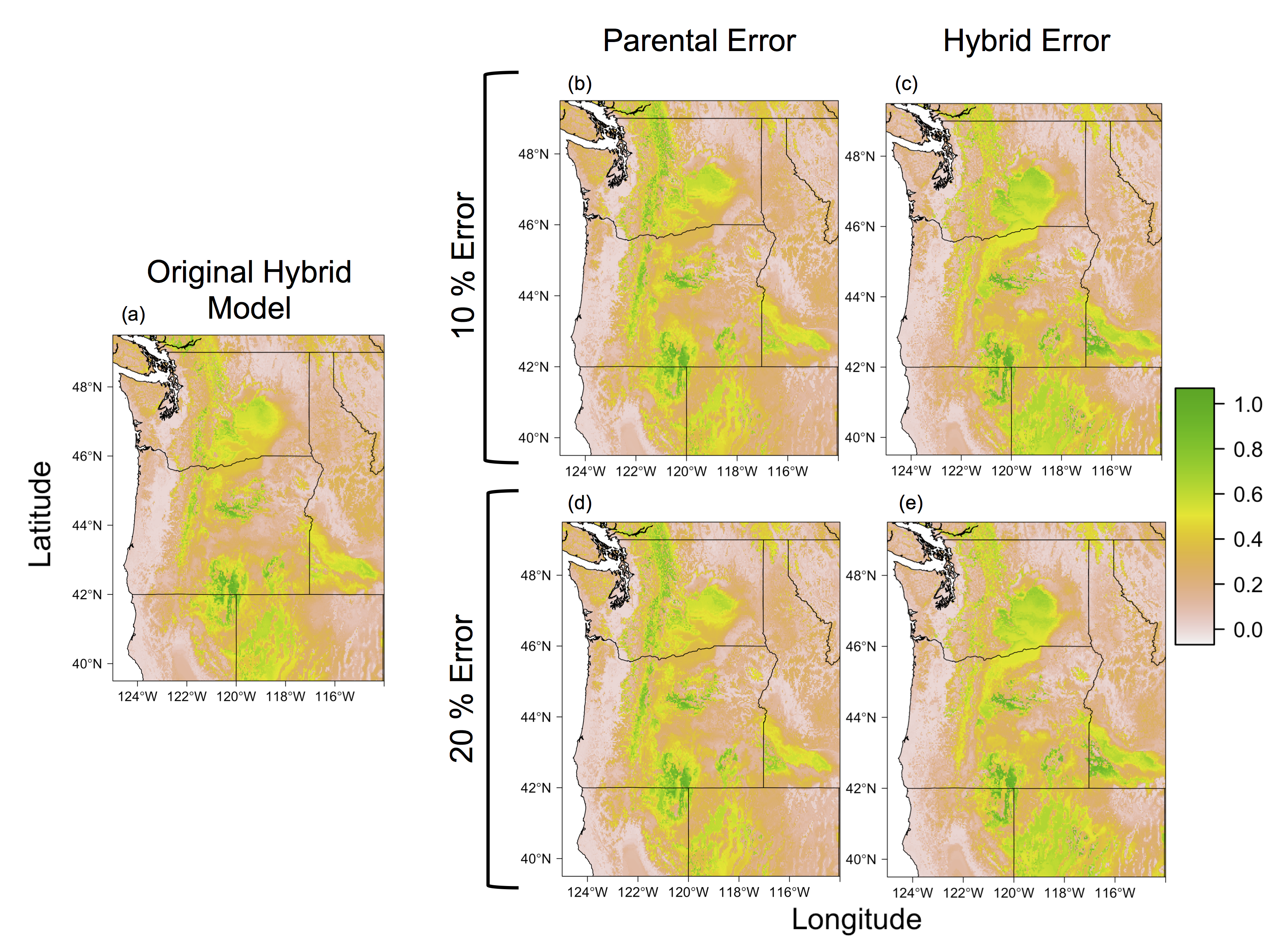

Supplement: Supplementary file 3 [file ECE3-6-7976-s003.tiff]

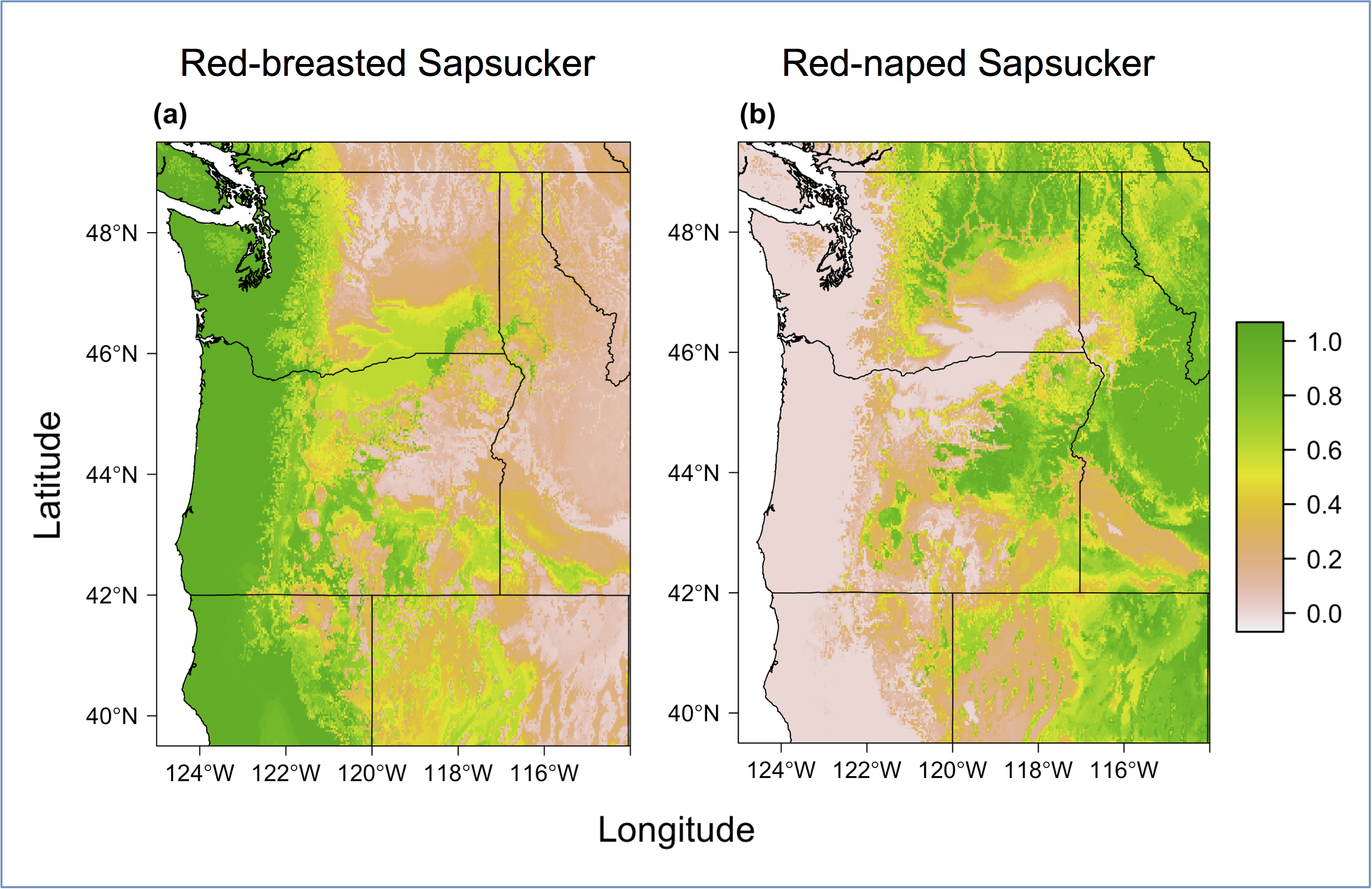

Supplement: Supplementary file 4 [file ECE3-6-7976-s004.tiff]
